# Long-term prevalence follow-up (1967–2022) of HTLV-2 among vulnerable indigenous populations in the Amazon region of Brazil

**DOI:** 10.3389/fmicb.2023.1217134

**Published:** 2023-06-22

**Authors:** Ricardo Ishak, Marluísa de Oliveira Guimarães Ishak, Isabella Nogueira Abreu, Luiz Fernando Almeida Machado, Sandra Souza Lima, Maria Alice Freitas Queiroz, Izaura Maria Cayres-Vallinoto, João Farias Guerreiro, Antonio Carlos Rosário Vallinoto

**Affiliations:** ^1^Laboratory of Virology, Institute of Biological Sciences, Federal University of Pará, Belém, Pará, Brazil; ^2^Postgraduate Program in Biology of Infectious and Parasitic Agents, Institute of Biological Sciences, Federal University of Pará, Belém, Pará, Brazil; ^3^Laboratory of Human and Medical Genetics, Institute of Biological Sciences, Federal University of Pará, Belem, Pará, Brazil

**Keywords:** HTLV-1/2, Amazon, indigenous, epidemiology, vulnerable population

## Abstract

**Introduction:**

Human T-lymphotropic virus 2 (HTLV-2) has been described for more than 30 years as an endemic infection in Brazilian indigenous populations, with its occurrence varying by age and sex, maintained mainly by sexual intercourse and mother-to-child transmission, favoring intrafamilial aggregation.

**Methods:**

The epidemiological scenario of HTLV-2 infection has been described among communities of the Amazon region of Brazil (ARB), with the number of retrospective positive blood samples increasing for more than 50 years.

**Results:**

Five publications were selected that showed the presence of HTLV-2 in 24 of 41 communities; the prevalence of infection was described among 5,429 individuals at five points in time. Among the Kayapó villages, the prevalence rates were described according to age and sex and reached up to 41.2%. Three communities (Asurini, Araweté, and Kaapor) were kept virus free for 27 to 38 years of surveillance. Low, medium and high prevalence levels of infection were defined, and two pockets of high endemicity were shown in the state of Pará, pointing to the Kikretum and Kubenkokrê Kayapó villages as the epicenter of HTLV-2 in the ARB.

**Discussion:**

The prevalence rates over the years have shown a decline among the Kayapó (from 37.8 to 18.4%) and an apparent change to a higher prevalence among females, but not during the first decade of life, usually associated with transmission from mother to child. Sociocultural and behavioral aspects, as well as public health policies directed toward sexually transmitted infections, might have positively influenced the decline in HTLV-2 infections.

## 1. Introduction

Human T-lymphotropic virus (HTLV) has been described for more than 40 years (Poiesz et al., 1980; Kalyanaraman et al., 1982; Gallo, 2005), and it was soon shown that the Amazon region of Brazil (ARB) was an important epidemiological geographical area for HTLV-2 (Maloney et al., 1992; Ishak et al., 1995). High endemicity was initially shown to occur among indigenous people and was largely distributed within the geographical area of eight different states (Maloney et al., 1992; Ishak et al., 1995). Contrary to the high prevalence of HTLV-1, commonly found among original peoples of Australia (Bastian et al., 1993; Einsiedel et al., 2016), the high endemicity in the ARB is mainly due to HTLV-2 infections among vulnerable populations (Braço et al., 2019; Abreu et al., 2023). Although HTLV-2 is not a common pathogen, its pathological role is still uncertain, and cases of disease associated with HTLV-2 are frequently reported (Kalyanaraman et al., 1982; Hjelle et al., 1992; Zucker-Franklin et al., 1992; Maytal et al., 1993; Peters et al., 1999; Araujo and Hall, 2004; Rosadas et al., 2014; Brasil, 2021; Gonçalves et al., 2022).

The initial reports of HTLV among indigenous people were equivocally reported as HTLV-1 (Nakauchi et al., 1990). High prevalence rates were reported, but they were related to HTLV-2 (Ishak et al., 1995; Vallinoto et al., 2002). Surveillance of HTLV-1/2 continued for the following 40 years, including visits to new villages and revisits to infected and noninfected villages (Vallinoto et al., 2002, 2019; Braço et al., 2019; Abreu et al., 2023). With the massive amount of information, it was possible to define clear patterns of HTLV-2 transmission within epidemiologically closed and semiclosed communities, mother-to-child transmission, intrafamiliar aggregation, possible clinical outcomes, and common pitfalls in the laboratory diagnosis of HTLV-2, possibly as a consequence of a new molecular subtype (HTLV-2c) inherent to the ARB (Ishak et al., 1995, 2001, 2007; Lewis et al., 2000; Vallinoto et al., 2002; da Costa et al., 2013; Braço et al., 2019; Abreu et al., 2023).

Most reports have shown heterogeneous HTLV-2 infection among the distinct indigenous groups in the ARB, with seroprevalence rates ranging from 0 to 40% (Maloney et al., 1992; Ishak et al., 1995, 2020; Vallinoto et al., 2019; Abreu et al., 2023). In the present study, possible epidemiological changes were analyzed within the scenario of HTLV-2 infection as a result of more than 30 years of studies in indigenous villages in the ARB and spanning 56 years of retrospectively investigated samples in an attempt to answer important questions: Why have seroprevalence rates seemed to drop over the years? What public health intervention was implemented, and which biological and behavioral aspects could explain this epidemiological scenario?

## 2. Materials and methods

### 2.1. Study design

The present study is a descriptive epidemiological approach aiming to evaluate the prevalence rate of HTLV-2 infection along the period ranging from 1967 to 2022. There are few studies regarding the presence of HTLV-2 among indigenous peoples from the Amazon region of Brazil and some of them used samples that were previously collected and stored for other purposes. In the absence of available database, there was a selection of five major criteria that would represent the best approaches for transversal studies to define prevalence rates, as listed below.

The prevalence level of infection in each community was rated as absent, low (prevalence range from 0.1 to 5%), medium (5.1 to 10%) or high (greater than 10.1%). The studies included in the present evaluation were those that (i) were clearly designed to provide global prevalence information on HTLV-2 in the ARB among indigenous communities (epidemiologically open, semiclosed or closed populations); (ii) included revisits to previously evaluated indigenous villages to provide two/three-point prevalence information; (iii) allowed the comparison of prevalence rates according to age and sex in a two-point prevalence investigation; (iv) used well defined antibody detection methods (Murex HTLV-I + II, DiaSorin, Dartford, United Kingdom), with confirmation of the responses (HTLV Blot 2.4 kit MP Diagnostics, Singapore, Republic of Singapore or INNO-LIA HTLV I/II Score, Fujirebio, Japan or real time PCR), for the purpose of presenting a strong seroepidemiological study; and (v) presented a reasonable sample in the original article.

### 2.2. Statistical analysis

Prevalence rates were defined by the number of individuals with the specified characteristics at a given point in time over the total number of individuals examined. Demographic information was collected from the articles included in this study for descriptive statistical analyses. Chi-square tests and Fisher’s exact tests were used to compare the general prevalence rates and the prevalence rates according to age and sex. BioEstat 5.3 (Ayres et al., 2007) was used for calculations, considering a significance level of 5% (*p* value <0.05).

## 3. Results

Among the published manuscripts that dealt with the prevalence of HTLV-2 among indigenous peoples from the ARB, five studies were selected on the basis of the defined criteria, particularly the consistency in the sampling and in the use of laboratory methods that were capable of distinguishing between seroreactivity to HTLV-1 and HTLV-2.

**Figure 1:**
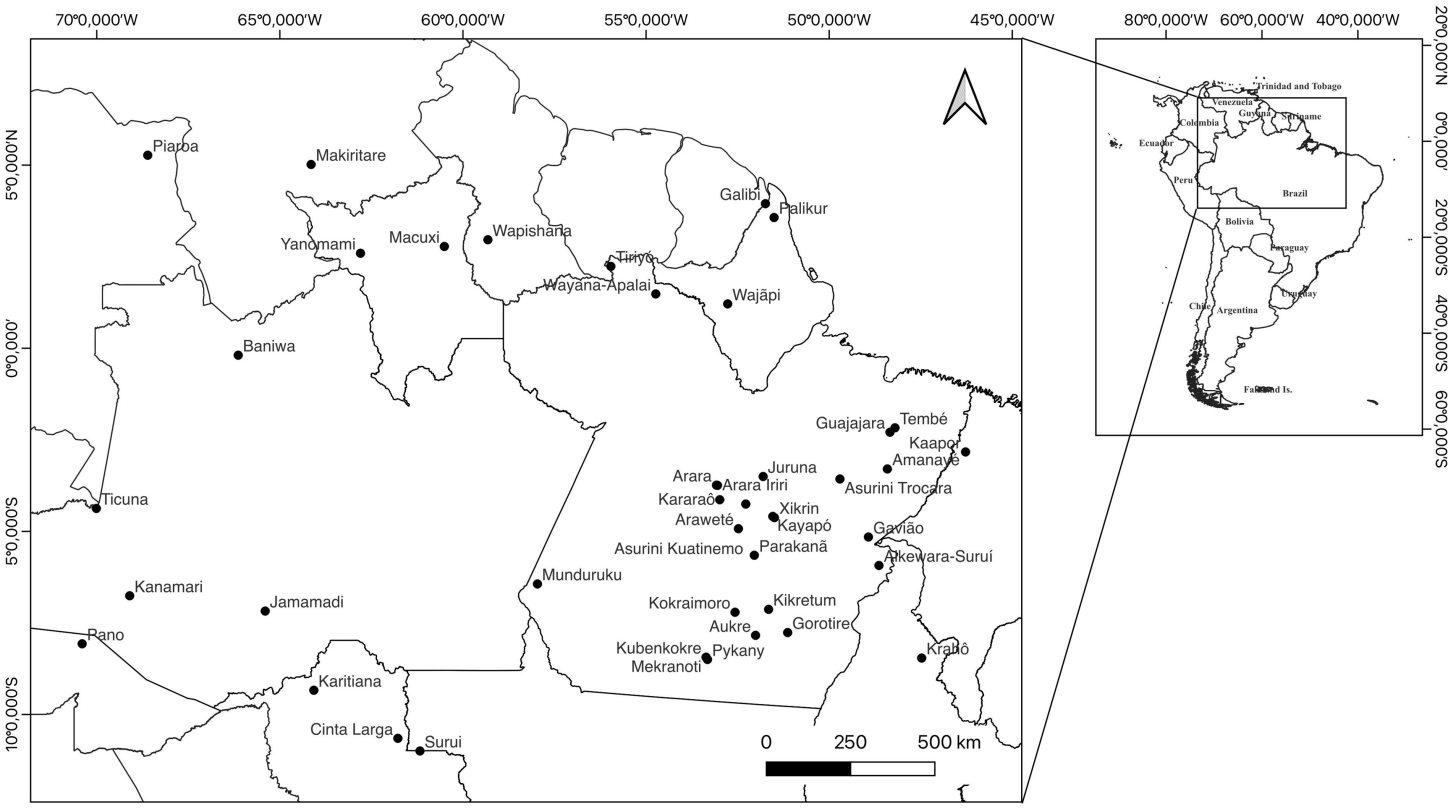
Geographical distribution of HTLV-2 infections among indigenous communities of the Amazon region of Brazil.

Table 1 shows the information from each study, comprising date of collection and publication, number of people examined, number of positive individuals and the communities investigated. The information included 5,429 individuals from 41 villages distributed within eight states of the ARB. The samples were obtained from 1966 to 2022 (an investigation spanning approximately 56 years). Figure 1 shows the approximate geographical situation of the indigenous communities within the ARB.

**Table 1:** Prevalence rates among indigenous communities of the Amazon region of Brazil sampled from 1966 to 2022.

	1992 (#1)				1995 (#2)		2002 (#3)			2019 (#4)			2023 (#5)		
	Year of collection	Cases/total	%	Year of collection	Cases/total	%	Year of collection	Cases/total	%	Year of collection	Cases/total	%	Year of collection	Cases/total	%
Maranhão															
Urubu Kaapor				1994	0/50	0.0							2021	0/21	0.0
Guajajara													2021	1/545	1.8
Amapá															
Galibi				1988	3/148	2.0									
Palikur				1988	0/38	0.0									
Wayapi				1994	1/71	1.4									
Pará															
Tembé													2021	0/186	0.0
Wayana Apalai				1993	1/50	2.0									
Tiryió				1990	4/26	15.4									
Asurini Trocara Kuatinemo				1984	0/100	0.0									0*
Arara Laranjal				1991	5/44	11.4									
Arara Kurambê				1991	0/2	0.0									
Arara Iriri				1991	0/28	0.0									
Araweté				1983	0/50	0.0							2021	0/468	0.0
Parakanã				1995	1/52	1.9							2021	0/211	0.0
Munduruku				1988	13/161	8.1							2020	2/317	0.6
Aikewara Surui													2022	1/215	0.5
Juruna													2021	0/100	0.0
Amanayé													2021	0/74	0.0
Gavião Parkateje													2022	1/252	0.4
Kararaô**				1986	6/23	26.1	2000	3/24	12.5				2021	1/39	2.6
Aukre**				1989	4/12	33.3									
Kubenkokre**				1990	40/107	37.4							2021	92/398	23.1
Pykany**					6/30	20.0									
Kikretum**					7/17	41.2									
Kokraimoro**					4/18	22.2							2021	16/140	11.4
Xikrin**										2015	29/263	11.0	2021	55/401	13.7
Gorotire**													2021	105/474	22.2
Kayapó**	1974	17/45	37.8												
Tocantins															
Krahô	1974	9/37	24.3												
Roraima															
Yanomami				1990	4/102	3.9									
Yanomami Venezuela	1966	1/150	0.6												
Macushi	1971	0/50	0												
Rondônia															
Cinta Larga				1987	1/50	2									
Surui				1987	0/57	0									
Karitiana				1986	2/50	4									
Amazonas															
Jamamadi				1986	2/36	5.6									
Baniwa	1976	0/48	0												
Ticuna	1976	0/49	0												
Kanamari	1976	0/47	0												
Acre															
Pano (Katukina)	1976	0/44	0												
General Prevalence		27/470	5.7		104/1322	7.9		3/24	12.5**		29/263	11.0**		274/3350	8.1
Prevalence in the Kayapó**		26/82	31.7		67/207	32.4		3/24	12.5		29/263	11.0		268/1452	18.4

(#1) Maloney et al., 1992; (#2) Ishak et al., 1995; (#3) Vallinoto et al., 2002; (#4) Braço et al., 2019; (#5) Abreu et al., 2023.

*Vallinoto et al., 2019; **Kayapó villages.

As listed in Table 1, several indigenous groups from the ARB were found to be free of HTLV-1/2 infection, including the Palikur (in the state of Amapá), Arara do Kurambê, Arara Iriri, Tembé, Juruna, Amanaye (in the state of Pará), and Surui (in the state of Rondônia). Several villages of indigenous groups were revisited during the period, and some of them remained HTLV-1/2 free, including the Kaapor (range of 27 years between visits), Asurini (35 years) and Araweté (38 years). In 1995, the Parakanã were visited a few weeks following their initial contact, and there was one single person found to be infected with HTLV-2, but none was detected 26 years later.

The general prevalence rates of HTLV-2 infection ranged from 5.7% (Maloney et al., 1992) to 8.1% (Abreu et al., 2023). There were nine villages that had a low (0.1–5%) prevalence of infection (Yanomami, Galibi, Waiãpi, Cinta Larga, Karitiana, Guajajara, Aikewara Surui, Wayana Apalai, and Gavião Parkateje), one with a medium (5.1–10%) prevalence of infection (Jamamadi), and nine with a high (>10.1%) prevalence of infection (Tiryio, Arara do Laranjal, and seven Kayapó villages: Aukre, Kubenkokre, Pykany, Kikretum, Kokraimoro, Xikrin, and Gorotire). Two groups, the Munduruku and the Kararaô, were regarded as having medium and high prevalence rates (8.1 and 26.1%, respectively) when examined in the 1980s but were reclassified as having low prevalence rates (Munduruku, 0.6% when revisited in 2020; Kararaô, 12.5% in 2000 and 2.6% in 2021).

The prevalence rates among the Kayapó villages ranged from 11.0% (Braço et al., 2019) to 37.8% (Maloney et al., 1992), showing a significant difference in the prevalence of infection (*p* < 0.0001). The highest rates were found among the Kikretum (41.2%) and Kubenkokrê (37.4%) in the 1990s and the Kubenkokrê (23.1%) in 2021.

Seroreactivity to HTLV-1 was sporadically reported (Ishak et al., 1995) and present in the Galibi (one person), Yanomami (three people), and Aukre Kayapó village (one person) in samples collected in the 1980s and 1990s. In the 2020s, HTLV-1 infections were present solely among four individuals of the Juruna people and Gorotire Kayapó village (Abreu et al., 2023).

**Figure 2:**
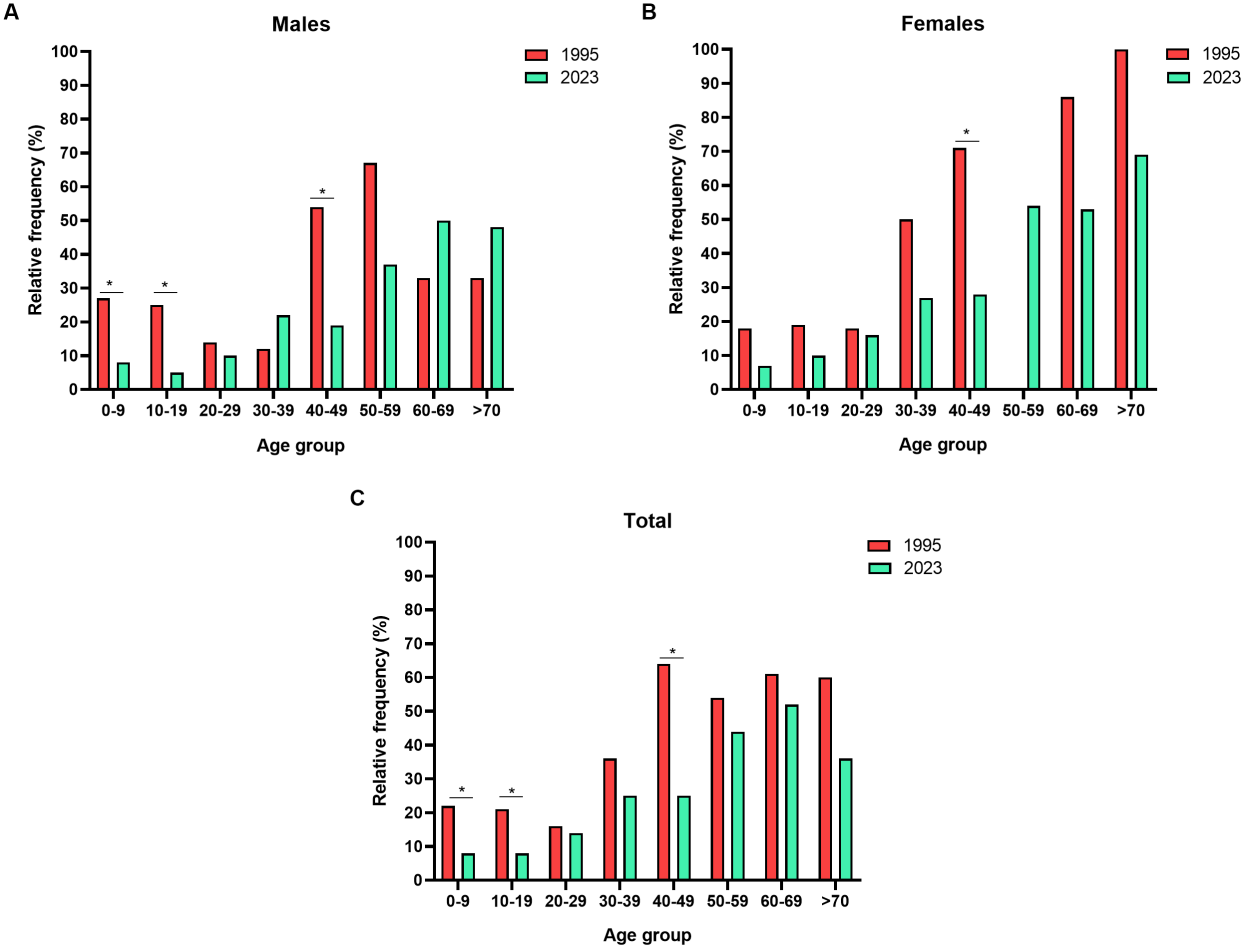
Prevalence rates among Kayapó communities of the Amazon region of Brazil according to age and sex groups. **(A)** Males, **(B)** Females and **(C)** Total (males plus females). Data obtained from Ishak et al. (1995) and Abreu et al. (2023). *p < 0.05.

The distribution of HTLV-2 according to age and sex among the Kayapó villages is shown in Table 2 and Figure 2, in which the differences in figures for more than 25 years are presented. Three levels of comparison were used: between males, between females and a third comparison adding males and females from two studies. The comparison of prevalence rates between males (31.4% vs. 16%; *p* = 0.0009), females (34.2% vs. 21.1%; *p* = 0.0025) and their sum in both studies (33% vs. 17.7%; *p* < 0.0001) was statistically significant.

**Table 2:** Prevalence rates among Kayapó communities of the Amazon region of Brazil according to age and sex.

Age	1995 (#1)			2023 (#2)			Total (1995 + 2023)			*p* value
	*n*	POS	%	*n*	POS	%	*n*	POS	%	
Males										
0–9	15	4	26.7	129	10	7.8	144	14	9.7	0.0412
10–19	20	5	25.0	118	6	5.1	138	11	8.0	0.0095
20–29	14	2	14.3	110	11	10.0	124	13	10.5	0.6344
30–39	8	1	12.5	85	19	22.4	93	20	21.5	0.6824
40–49	11	6	54.5	52	10	19.2	63	16	25.4	0.0391
50–59	9	6	66.7	43	14	32.6	52	20	38.5	0.1246
60–69	6	2	33.3	8	4	50.0	14	6	42.8	0.6270
>70	3	1	33.3	42	20	47.6	45	21	46.7	1.000
Total	86	27	31.4	587	94	16.0	673	121	18.0	0.0009
Females										
0–9	22	4	18.2	159	12	7.5	181	16	8.8	0.1108
10–19	42	8	19	184	18	9.8	226	26	11.5	0.1527
20–29	11	2	18.2	192	31	16.1	203	33	16.2	0.9811
30–39	14	7	50	100	27	27.0	114	34	29.8	0.1471
40–49	14	10	71.4	89	25	28.1	39	35	89.7	0.0043
50–59	2	0	0.0	46	25	54.3	48	25	52.1	0.2243
60–69	7	6	85.7	17	9	52.9	24	15	62.5	0.1907
>70	2	2	100	39	27	69.2	41	29	70.7	0.5756
Total	114	39	34.2	826	174	21.1	940	213	22.6	0.0025
Total (males and females)										
0–9	37	8	21.6	288	22	7.6	325	30	9.2	0.0137
10–19	62	13	21.0	302	24	7.9	364	37	10.2	0.0042
20–29	25	4	16.0	302	42	13.9	327	46	14.1	0.7613
30–39	22	8	36.4	185	46	24.9	207	54	26.1	0.3658
40–49	25	16	64	141	35	24.8	166	51	30.7	0.0002
50–59	11	6	54.5	89	39	43.8	100	45	45.0	0.7328
60–69	13	8	61.5	25	13	52.0	38	21	55.3	0.8281
>70	5	3	60.0	81	29	35.8	86	32	37.2	0.3562
Total	200	66	33.0	1,413	250	17.7	1,613	316	19.6	< 0.0001

#1 (Ishak et al., 1995); #2 (Abreu et al., 2023).

The same approach was used to compare the prevalence rates during the first 9 years of life to have an approximate figure to indicate *in utero* or breastfeeding transmission. Significant differences were found when comparing males (26.7% vs. 7.8%; *p* = 0.0412) between both studies but not females (18.2% vs. 7.5%; *p* = 0.1108). The third comparison adding males and females from the two studies (21.6% vs. 7.6%) was also significantly different (*p* = 0.0137) from the comparison in the initial study published.

## 4. Discussion

The origin of HTLV-2 infection in the ARB, was previously proposed to be in close association with the intense migration of humans who crossed the Behring Strait and arrived in South America (Ishak et al., 2020). The search for HTLV-1/2 among indigenous communities in the ARB dates back to the 1980s, but it was not successful in detecting HTLV (Andrada-Serpa et al., 1988) or specifying seroreactivity to HTLV types (Nakauchi et al., 1990). By that time, the use of equivocal and crude assays was not designed to detect infection with HTLV-2 (Maloney et al., 1992). Furthermore, there was soon a new molecular subtype of the virus, named HTLV-2c, that was characteristic of the ARB and contributed to the difficulties in detecting HTLV-2 (Ishak et al., 1995, 2007; Vallinoto et al., 2002).

HTLV-2 is widely distributed in the ARB, but upon closer inspection, it is geographically distributed mainly within two pockets in the state of Para (Figure 1). The peripheral areas of the region show the absence of the virus, as shown in the studies by Maloney et al. (1992), Ishak et al. (1995), and Abreu et al. (2023). The studies described in Table 1 investigated 5,429 individuals, and HTLV-2 was found within 24 (58.5%) out of 41 communities. Several villages were free of infection, and at least three (Asurini, Araweté and Kaapor) were sequentially followed up and showed the absence of the virus ranging from 27 to 38 years apart (Vallinoto et al., 2019; Abreu et al., 2023).

Upon revisiting the Parakanã village, the virus was not present. It is possible that the sole person who was found to be positive in 1995 was not successful in further transmitting the virus. Although the Asurini, Araweté and Parakanã are geographically linked to one of these two major pockets of high endemicity of HTLV-2, it seems that the absence of contact among them, as a consequence of disputes for different reasons, was a sufficient containment measure that prevented dissemination of the virus (Vallinoto et al., 2019).

Whatever reasons that maintain the absence of HTLV-2 among the Kaapor, Palikur, Tembé, Asurini, Arara, Araweté, Parakanã, Juruna, Amanayé, Macushi, Surui, Baniwa, Ticuna, Kanamari and Pano should be investigated, and public health policies should be strongly recommended to maintain indigenous communities free from HTLV-1/2 infection.

In contrast, low, middle, and high prevalence rates of HTLV-2 were observed among the Kayapó villages. Although HTLV-2 has been detected in eight states of the ARB, the vast majority of infected communities were located in the state of Pará, and the epicenter of the virus seemed to be within the Kayapó villages Kubenkokrê and Kokraimoro, which had the highest numbers of infections during two sequential visits. However, in the last visit (Abreu et al., 2023), Gorotire village, which was visited for the first time, showed a high endemicity of the virus.

Continuous visits to the indigenous villages of the ARB starting in the 1980s, with a major and main objective of providing access to medical personnel, health aid and treatment coupled with surveillance of infectious diseases and vaccination coverage, showed us that a clearer clinical epidemiological picture is being depicted. Although the prevalence rates are still high in the two major pockets of hyperendemicity of HTLV-2, it seems that the prevalence of HTLV-2 has clearly declined from when the first samples were collected in 1966 (Maloney et al., 1992) to 2022 (Abreu et al., 2023), but which public intervention was implemented to lead to such a situation? The Brazilian Ministry of Health is constantly putting forward campaigns aimed at the prevention of sexually transmitted infections among the different indigenous communities of the ARB. It is reasonable to believe that the distribution of condoms or other prevention approaches could have contributed to the decline in HTLV-2 together with other STIs.

The recent observation of the distribution of HTLV-2 according to age and sex still supports the need to formulate public policies to prevent virus infection through two of the most important routes of transmission (Abreu et al., 2023). Prevalence rates among children up to 9 years of age are borderline high and are clearly linked to transmission either *in utero* or by breastfeeding. It is important to emphasize that in some cultures, cross-breastfeeding is a common cultural practice that positively impacts the spread of the virus among the individuals of the community, as an HTLV-infected mother is able to infect other children apart from their own. Family aggregation is a common event (Ishak et al., 1995, 2001; da Costa et al., 2013), and more recently, with a larger sampling, it was possible to show that there was no difference between prevalence rates associated with sex in the first years of life (Abreu et al., 2023), which apparently favors transmission by chance, *in utero*, perinatally or by breastfeeding.

A second wave of infection seemed to be associated with sexual transmission between young adults older than 20 years of age. The sudden rise in prevalence rates among males and females, from approximately 10 to 26%, is epidemiological evidence of the change in the main route of transmission of HTLV-1 in communities.

Biological changes in the virus could also contribute to this apparently new epidemiological scenario, but there is no further evidence to support that HTLV-2c has changed since it was initially reported (Ishak et al., 1995), including the conserved nature of the Tax protein from strains collected over time (Lewis et al., 2000; Lopes, 2007). Behavioral aspects regarding the virus during the investigation period showed few changes apart from the higher prevalence among women, which was not observed at first.

Most reports have shown heterogeneous HTLV-2 infections among distinct indigenous groups in the ARB, with seroprevalence rates ranging from 0 to 40% (Ishak et al., 1995, 2020; Vallinoto et al., 2019; Abreu et al., 2023). The subject has been previously addressed, and there were several problems associated with the design of epidemiological studies in the ARB, showing clear differences in their findings in both urban and nonurban population groups (Ishak et al., 2020). Regarding indigenous communities, this seems to be the result of different sociocultural behaviors as well as geographic isolation. These ethnographic aspects, added to the vast geographic area of Amazonia, make it difficult to implement public health policies to block HTLV transmission among vulnerable original indigenous peoples of the Amazon region (Ishak et al., 2020).

Finally, it is relevant to mention that the presence of HTLV-1 is quite rare, as observed among nine individuals of the Galibi, Aukre, Yanomami, Juruna and Gorotire villages (Ishak et al., 1995; Abreu et al., 2023). Their geographical situation is quite different, and it is possible that contact with outsiders may have taken the virus either within the indigenous communities in the recent past or that their contamination occurred during visits to urban areas of the ARB.

## 5. Concluding remarks

Since the 1990s the Virus Laboratory associated with the Laboratory of Medical Genetics and FUNAI (the main government authority in Brazil) to provide medical assistance in regard to genetic and chronic diseases, as well as bacterial and virus infections. Short visits (10–15 days) were arranged with a medical and research team to investigate the presence of maladies and to provide further assistance in the prevention and control of diseases, whenever possible. Prevention of infectious agents is a difficult task to achieve when it comes to sexual and breastfeeding transmission. According to their needs, several villages of indigenous groups were usually revisited to evaluate the clinical and epidemiological needs.

The search for information regarding HTLV among indigenous populations was sometimes frustrating because of the small number of publications available. This limitation is a possible consequence of the authorities’ restrictions imposed to access the communities, the difficulties to reach most villages that are in geographical isolated areas and make it even more difficult to prepare prospective studies. Considering all these adverse conditions, the choice to prepare prevalence studies with multiple points of observation in time is the best option to evaluate the actual changes in the frequency of HTLV and other viral and bacterial infections.

## Data availability statement

The raw data supporting the conclusions of this article will be made available by the authors, without undue reservation.

## Author contributions

All authors listed have made a substantial, direct, and intellectual contribution to the work and approved it for publication.

## Funding

This work was supported by the Conselho Nacional de Desenvolvimento Científico e Tecnológico, CNPq (Research grants: RI #312979/2018–5, AV #302935/2021–5, LM #314209/2021–2, MQ #304835/2022–6, and JG#311097/2019–7).

## Conflict of interest

The authors declare that the research was conducted in the absence of any commercial or financial relationships that could be construed as a potential conflict of interest.

## Publisher’s note

All claims expressed in this article are solely those of the authors and do not necessarily represent those of their affiliated organizations, or those of the publisher, the editors and the reviewers. Any product that may be evaluated in this article, or claim that may be made by its manufacturer, is not guaranteed or endorsed by the publisher.
